# Cold-Induced Reprogramming of Subcutaneous White Adipose Tissue Assessed by Single-Cell and Single-Nucleus RNA Sequencing

**DOI:** 10.34133/research.0182

**Published:** 2023-06-28

**Authors:** Qing Liu, Qiaoyun Long, Jiayu Zhao, Wenjie Wu, Zexin Lin, Wei Sun, Ping Gu, Tuo Deng, Kerry Martin Loomes, Donghai Wu, Alice P. S. Kong, Jingying Zhou, Alfred S. Cheng, Hannah Xiaoyan Hui

**Affiliations:** ^1^ School of Biomedical Sciences, The Chinese University of Hong Kong, Hong Kong, China.; ^2^ Department of Pharmacology and Pharmacy, The University of Hong Kong, Hong Kong, China.; ^3^ Guangzhou Institute of Biomedicine and Health, Chinese Academy of Sciences, Guangzhou, China.; ^4^ China-New Zealand Joint Laboratory on Biomedicine and Health, Chinese Academy of Sciences, Guangzhou, China.; ^5^ Department of Endocrinology, Jinling Hospital, Nanjing University, School of Medicine, Nanjing, China.; ^6^ National Clinical Research Center for Metabolic Diseases, and Department of Metabolism and Endocrinology, The Second Xiangya Hospital of Central South University, Changsha, China.; ^7^ Key Laboratory of Diabetes Immunology, Ministry of Education, and Metabolic Syndrome Research Center, The Second Xiangya Hospital of Central South University, Changsha, China.; ^8^ Clinical Immunology Center, The Second Xiangya Hospital of Central South University, Changsha, China.; ^9^ School of Biological Sciences and Maurice Wilkins Centre, University of Auckland, Auckland, New Zealand.; ^10^ Department of Medicine and Therapeutics, The Chinese University of Hong Kong, Hong Kong, China.; ^11^ Hong Kong Institute of Diabetes and Obesity, The Chinese University of Hong Kong, Prince of Wales Hospital, Shatin, Hong Kong, China.; ^12^ Li Ka Shing Institute of Health Sciences, The Chinese University of Hong Kong, Prince of Wales Hospital, Shatin, Hong Kong, China.

## Abstract

Adipose browning has demonstrated therapeutic potentials in several diseases. Here, by conducting transcriptomic profiling at the single-cell and single-nucleus resolution, we reconstituted the cellular atlas in mouse inguinal subcutaneous white adipose tissue (iWAT) at thermoneutrality or chronic cold condition. All major nonimmune cells within the iWAT, including adipose stem and progenitor cells (ASPCs), mature adipocytes, endothelial cells, Schwann cells, and smooth muscle cells, were recovered, allowing us to uncover an overall and detailed blueprint for transcriptomes and intercellular cross-talks and the dynamics during white adipose tissue brown remodeling. Our findings also unravel the existence of subpopulations in mature adipocytes, ASPCs, and endothelial cells, as well as new insights on their interconversion and reprogramming in response to cold. The adipocyte subpopulation competent of major histocompatibility complex class II (MHCII) antigen presentation is potentiated. Furthermore, a subcluster of ASPC with CD74 expression was identified as the precursor of this MHCII^+^ adipocyte. Beige adipocytes are transdifferented from preexisting lipid generating adipocytes, which exhibit developmental trajectory from de novo differentiation of amphiregulin cells (Aregs). Two distinct immune-like endothelial subpopulations are present in iWAT and are responsive to cold. Our data reveal fundamental changes during cold-evoked adipose browning.

## Introduction

The old view that white adipose tissue (WAT) is a simple and inert organ for energy storage has been revolutionized in the past decades [1]. In addition to storing extra energy in the form of neutral lipid in mature adipocytes, adipose tissue is regarded as a metabolically active endocrine organ, from which a myriad of bioactive molecules are secreted that affect metabolism in endocrine, paracrine, and autocrine manners [1,2]. In addition to mature adipocytes, WAT is composed of multiple cell populations, including adipose stem and progenitor cells (ASPCs), endothelial cells, smooth muscle cells (SMCs), various immune cells, and Schwann cells as the component of the peripheral nervous system. As a critical regulator in systemic energy homeostasis, WAT is highly plastic in that it undergoes various cellular and structural remodeling processes, including expansion in adipocyte size (hypertrophy) and number (hyperplasia), recruitment of immune cells, and remodeling of the vasculature and the extracellular matrix (ECM), all of which coordinate to accommodate adequate tissue expansion, oxygenation, and mobilization of nutrients [3].

Single-cell RNA sequencing (scRNA-seq) allows high-throughput profiling of transcriptomic signatures one cell at a time, leading to a better appreciation on the diversity of adipose cell populations [4]. Using scRNA-seq, a number of studies have delineated the transcriptome of the stroma vascular cell (SVC) populations in different adipose depots [5–13]. In addition, a burgeoning number of studies have used single-nucleus RNA sequencing (snRNA-seq) technology that overcomes the incompatibility of large-sized adipocytes with scRNA-seq to investigate the isolated adipocytes from brown adipose tissue (BAT) [14,15] and WAT [16–18]. In particular, snRNA-seq analysis on WAT has uncovered intriguing knowledge on the heterogeneity and plasticity of cell populations, as well as cell–cell communications under lean and obese conditions [16–18].

In addition to nutrition change-associated remodeling, conversion from a white to brown-like (now called beige) phenotype is another key manifestation of WAT plasticity [19]. Granneman and the colleagues [5] used scRNA-seq on mouse WAT to identify distinct subpopulations of adipocyte progenitors and immune cells with β_3_-adrenergic receptor agonism. Importantly, cold exposure appears not to be just a simple activation of β-adrenergic receptors since cold exposure, but not sympathomimetics, activates BAT activity without increasing blood pressure [20]. Furthermore, there exist β-adrenergic receptor-independent mechanisms that facilitate cold-induced adipose tissue remodeling [21]. Rajbhandari et al. [22] revealed a subpopulation of adipocyte enriched in lipid metabolism, norepinephrine signaling, and thermogenesis after a short-term cold challenge in mice. Interestingly, a recent study found that cold-activated BAT competes against tumors for glucose and thereby helps inhibit tumor growth [23], suggesting that cold exposure could be a promising cancer therapy. Therefore, a thorough understanding on the remodeling of cellular traits in WAT undergoing longer-term cold adaptation, which is more reminiscent under human conditions, is expected to provide insights on management of metabolic-related diseases and beyond, such as cancer.

Here, we used snRNA-seq and scRNA-seq to obtain cellular maps of mature adipocytes and nonimmune cells in mouse inguinal WAT (iWAT), the fat depot that is most responsive to cold-induced beiging, either with or without chronic cold stress. Our dataset reveals insights into the transcriptional reprogramming in WAT upon chronic cold-evoked changes at single-cell resolution in various cell types and subpopulations, including mature adipocytes, ASPCs, endothelial cells, SMCs, and Schwann cells. Furthermore, by analyzing the secretome and ligand–receptor pairing in a cell-type-specific manner, our finding sheds light on the exquisite networking between adipocytes and neighboring cell components and peripheral organs.

## Results

### snRNA-seq and snRNA-seq reveal differential cell types in iWAT under thermoneutrality and cold-adapted conditions

**Fig. 1.**
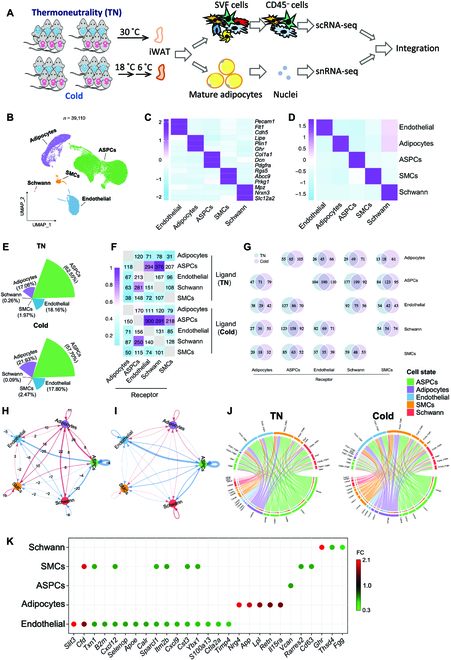
Cellular compositions and intercellular interactions in iWAT are altered by cold exposure. (A) Workflow of the experiment. Three male and 3 female C57BL/6J mice were either housed at thermoneutrality (TN; 30 °C) or put to cold temperature (18 °C for 14 days followed by 6 °C for 7 days). CD45^−^ cells from the SVF and the nuclei of the mature adipocytes were subjected to sc-seq and sc-seq, respectively. (B) UMAP of iWAT cell types. (C) Heatmap showing scaled average expression of selected cell-type-enriched marker genes. (D) Heatmap showing average scaled gene module scores for the top 50 most enriched expressed marker genes in each cluster. (E) The fraction of each cell type in iWAT from TN and cold-temperature-adapted mice. (F) Heatmap showing the number of interactions. (G) Venn diagrams showing the overlap of significant interactions between different types of cells in iWAT. (H and I) The number (H) and the relative strength (I) of the unique interactions after cold exposure. (J) Circos plots showing the Laminin-related ligand–receptor interactions. (K) Dot plot representing differential secretome components identified in TN and cold condition.

To generate a comprehensive cellular atlas involved in adipogenesis of iWAT at single-cell resolution and their adaptive response to chronic cold adaptation, we used 2 independent cohorts of C57BL/6J mice. To identify the gender-independent changes, in each cohort, we pooled tissue samples from male and female mice as done in a previous study [9]. Each cohort was either housed at 30 °C (thermoneutrality) or subjected to cold. scRNA-seq on nonimmune stromal vascular cells (CD45^−^) and snRNA-seq on nuclei from mature adipocytes were performed (Fig. 1A and Fig. S1). Comprehensive laboratory animal monitoring system (CLAMS) and seahorse analysis showed that oxygen consumption rates in whole body and in iWAT were significantly potentiated in mice under this cold challenge regimen (Fig. S2A and B), along with the appearance of multilocular cells typical for beige adipocytes (Fig. S2C).

After data preprocessing and quality control, 17,890 single cells and 22,220 single nuclei of high quality were obtained and integrated using the *FindIntegrationAnchors* and *IntegratedData* functions of the Seurat package. The different cells between thermoneutrality and cold conditions were well aligned, suggesting minimal batch effects (Fig. S3A). By unsupervised graph-based clustering, we detected 5 distinct clusters in the integrated atlas (Fig. 1B to D). On the basis of the highly variable genes of each cluster and classical marker genes, they were identified as ASPC (*Col1a1^+^*), adipocyte (*Plin1^+^*), endothelial cell (*Pecam^+^*), SMC (*Cacna1c^+^*), and Schwann cell (*Mpz^+^*), respectively (Fig. 1C, Fig. S3B, and Table S1). Adipocytes were primarily present in the snRNA-seq dataset, which is another piece of evidence for appropriate data integration (Fig. S3C). snRNA-seq dataset also includes ASPCs, and they exhibited differential expression patterns to those in scRNA-seq dataset, likely indicating that these ASPCs are lipid-laden and at a relatively later stage of differentiation compared to those cell pellets being collected for scRNA-seq analysis. SMCs and endothelial cells in the snRNA-seq are likely the contaminating cells since they largely overlap with the same cell types in the scRNA-seq dataset. By comparing the cell composition of each cluster in iWAT between thermoneutrality and cold condition, we found slightly lower proportions of ASPCs, Schwann cells, and endothelial cells relative to mature adipocytes and SMCs in cold-exposed iWAT (Fig. 1E). No obvious difference was found in the percentage of SMCs between cold and thermoneutral iWAT (Fig. 1E).

### Thermoneutrality and cold evoke differential intercellular communications in adipose tissue

Cell–cell interactions between the identified cell types within iWAT were examined by CellPhoneDB, which uses the expression of ligand–receptor pairs as a proxy for intercellular communications [24]. Potential cell–cell interactions were identified between all the cells in our database, demonstrating the operation of bidirectional cellular networks within iWAT (Fig. 1F, Fig. S3D, and Table S2). Unexpectedly, adipocytes displayed a relatively low number of communications to other cells at thermoneutrality, and, in particular, the lowest number of interactions was between adipocytes and SMCs where only 31 ligand–receptor pairs were identified (Fig. 1F and Table S2). In contrast, cold exposure strongly enhanced the intercellular communication between adipocytes and the other cell types (Fig. 1F and G). A total of 61 (66.3% of total number of detected pairs), 71(47.7%), 66 (48.2%), and 105 (46.7%) ligand–receptor pairs from adipocytes toward SMCs, Schwann cells, endothelial cells, and ASPCs were detected respectively (Fig. 1G and Table S2). Using CellChat, another tool for analysis of cell–cell communication, similar conclusion is obtained that it was mainly the interactions associated with the adipocytes that were increased both in number and in strength after cold exposure (Fig. 1H and I). For example, recognition of adipocyte-derived laminin signals by the integrin receptors from the other cell types was increased after cold exposure (Fig. 1J and Fig. S3D), which is a sign of ECM remodeling elicited by the adipocytes. In contrast, much of the cellular interactions among the other cells, especially from ASPC to endothelial cells and Schwann cells, were down-regulated (Fig. 1H and I). These findings suggest that the mature adipocytes serve as a central hub to positively coordinate the tissue remodeling, including intra-adipose neuronal network growth upon cold exposure.

### Cold exposure alters secretory features of adipose tissue

As a critical endocrine organ, WAT secretes a myriad of factors that are implicated in coordinating whole-body physiology in healthy and obese phenotypes. We identified differentially regulated secretory factors from each cell type in the context of thermoneutrality and cold (Fig. 1K and Table S3). Interestingly, there were more down-regulated versus up-regulated cytokines after cold stimulation. For example, *Nrg4* and *Lpl* were abundantly induced in adipocytes by cold, possibly to facilitate energy metabolism, neurite outgrowth, and substrate extraction. Unexpectedly, *App*, the secretory protein associated with Alzheimer’s disease [25], was also induced in adipocytes after cold exposure. In ASPCs, the expression of *Vcan*, which encodes a proteoglycan Versican and is found in the ECM, was suppressed upon cold adaptation. In addition, ECM-associated protein *Sparcl1* from endothelial cells and SMCs was also down-regulated by cold exposure, implying that reconstruction of the ECM in iWAT involves coordinated actions from different cells within the depot. *Slit3*, recently reported to increase sympathetic activity and thermogenesis in adipose tissue [26], was induced in endothelial cells, raising the possibility that endothelial cells also actively contribute to WAT browning in response to cold exposure.

### Cold exposure triggers higher lipid metabolism in amphiregulin cells

**Fig. 2.**
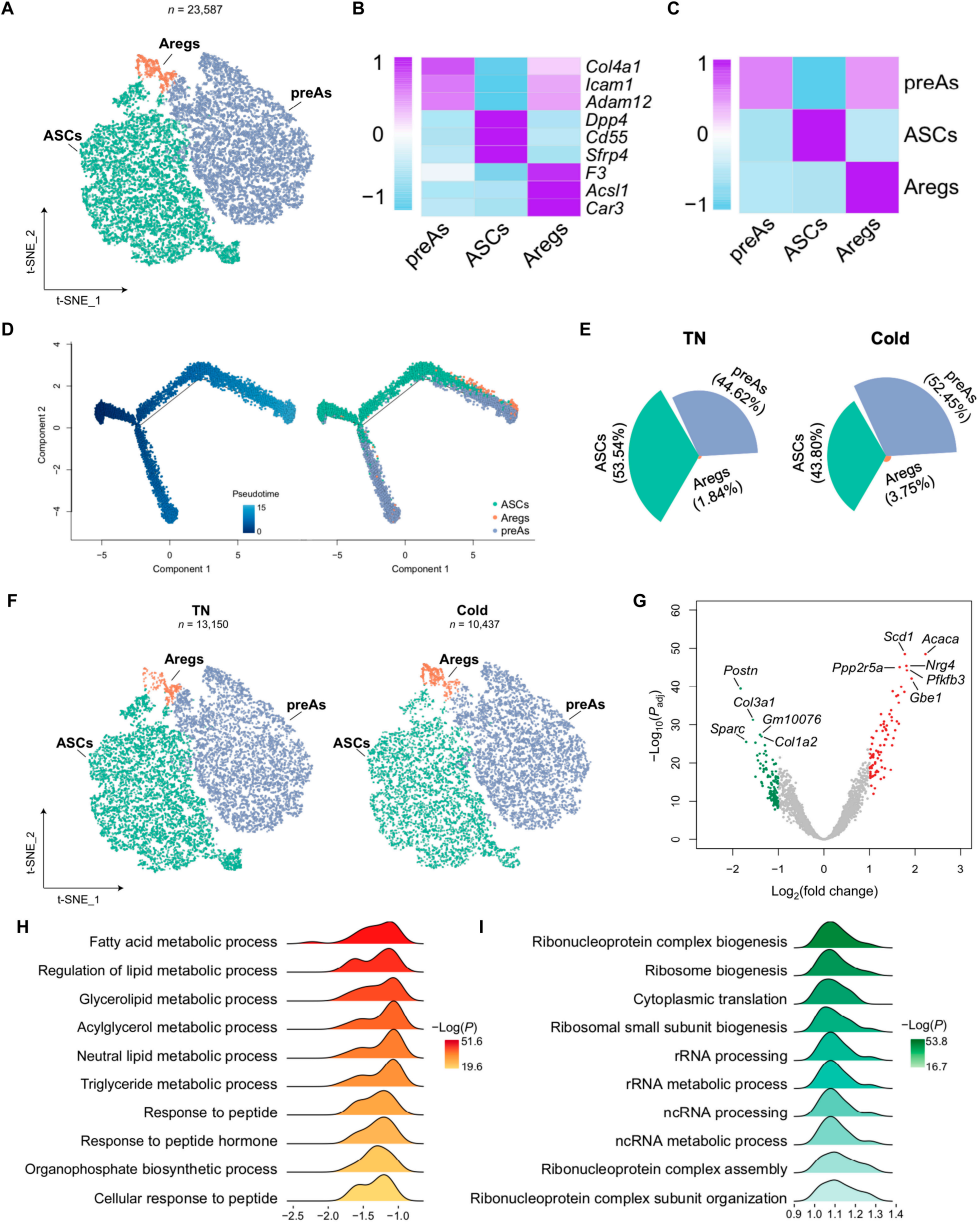
Lipid metabolism is potentiated in Areg cells after cold adaptation. (A) t-SNE of ASPC subpopulations. (B) Heatmap showing scaled average expression of selected cell-type-enriched marker genes. (C) Heatmap showing average scaled gene module scores for the top 50 most enriched expressed marker genes in each cluster. (D) The trajectory inference of ASPC subpopulations. (E) The fraction of each ASPC subpopulation in TN and cold-adapted mice. (F) t-SNEs of ASPC subpopulations in iWAT from TN and cold-adapted mice. (G) Volcano plot showing DEGs in Aregs (cold versus TN). (H and I) Top 10 most up-regulated (H) and down-regulated (I) pathways. rRNA, ribosomal RNA; ncRNA, noncoding RNA.

Reclustering of ASPCs resulted in 3 distinct subpopulations that were identified on the basis of the expression of classic cell type markers (Fig. 2A to C, Fig. S4A, and Table S4). These 3 subpopulations were annotated as adipocyte stem cell (ASC; *Dpp4^+^*), preadipocyte (preA; *Icam1^+^*), and amphiregulin cell (Areg) (*F3*^+^) (Fig. 2A to C and Fig. S4A), according to previous scRNA-seq studies [9,17]. Differentiation trajectory inference by Monocle2 demonstrated that both preAs and Aregs were derived from ASCs (Fig. 2D), consistent with previous findings [9].

ASPC composition was slightly changed by cold in that the proportion of ASCs in total ASPCs was decreased from thermoneutrality (53.54%) to cold (43.8%), while the relative proportion of preAs and Aregs was increased in cold stimulation (Fig. 2E and F), implying that the differentiation from ASC to preA and Areg is potentiated during cold exposure. Analysis of the transcriptional events in each ASPC subpopulation revealed that cold-induced substantial gene expression changes in Aregs, where 204 differentially expressed genes (DEGs; 84 up-regulated and 120 down-regulated) were found (Fig. 2G and Table S5). Pathway enrichment analysis uncovered that the increased DEGs in Aregs were enriched in the process of “fatty acid metabolic process”, “regulation of metabolic process”, and “glycerolipid metabolic process”, while the decreased DEGs were mainly enriched in the “ribonucleoprotein complex biogenesis”, “ribosome biogenesis”, and “cytoplasmic translation” process (Fig. 2H and I). In contrast, ASC and preA subsets displayed fewer numbers of DEGs (Fig. S4B and C). In ASCs, *Ccl2* was the only gene found up-regulated by cold, implicating its possible contribution to angiogenesis and remodeling of the local immune context (Table S5). In preAs, 100% of the DEGs were down-regulated including ECM-related genes (Fig. S4C and Table S5). These results indicate that compared to ASC and preA, the Areg subpopulation plays a primary role in contributing to the WAT remodeling process stimulated by cold.

### Distinct adipocyte subpopulations are present in iWAT

**Fig. 3.**
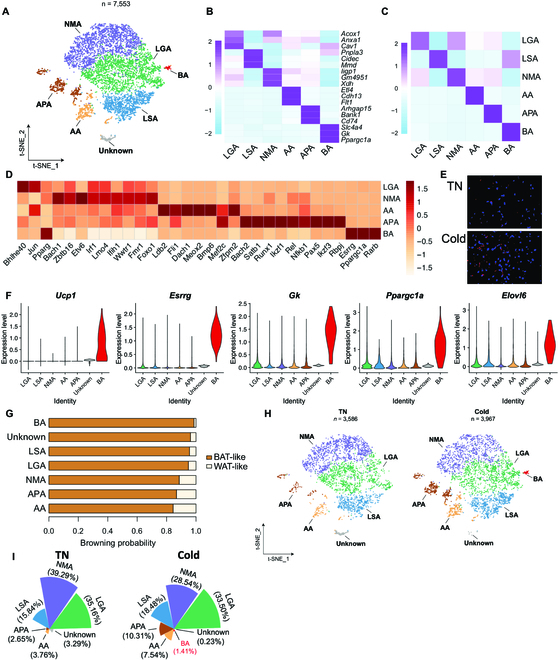
Adipocytes are subclustered to 6 subtypes in iWAT. (A) t-SNE of adipocyte subpopulations. (B) Heatmap showing scaled average expression of selected cell-type-enriched marker genes. (C) Heatmap showing average scaled gene module scores for the top 50 most enriched expressed marker genes in each cluster. (D) Top 10 TFs in subcluster adipocytes. (E) smFISH of *Elt4* (red) in mouse iWAT without or with cold. Tissue was also counter stained with 4′,6-diamidino-2-phenylindole (blue). (F) Violin plot of marker genes in adipocyte subclusters. (G) Browning potential of adipocyte subclusters. (H) t-SNEs of adipocyte subpopulations under TN and cold condition. (I) The fraction of each adipocyte subpopulation in TN and cold.

Mature adipocytes exist as distinct subpopulations in epididymal WAT and classical BAT [15–18]. To characterize adipocyte heterogeneity in subcutaneous WAT, we reclustered the adipocytes and detected 7 distinct subpopulations (Fig. 3A to C). All 7 subclusters are high in classical adipocyte markers, such as *Plin1/4*, *Pparg*, *Lipe*, *Lep*, and *Adrb3*, as compared with the remaining cells (Fig. S5A). These subpopulations were annotated on the basis of the highly variable genes of each cluster (Fig. S5B and C and Table S6). Two subpopulations express high levels of genes associated with lipid biosynthesis (such as *Acaca* and *Acly)* and lipid uptake, transport, and esterification (such as *Lpl*, *Pnpla3*, *Cidec*, and *Dgat2*) (Fig. S5D and E and Table S6). These observations are reminiscent of the lipogenic adipocytes (LGAs) and lipid scavenger adipocytes (LSAs) as detected by Sárvári et al. [17] in epididymal WAT. Transcription factor (TF) *Bhlhe40* was selectively enriched in LGAs (Fig. 3D), which is consistent with an important role in the control of lipid metabolism through binding to the peroxisome-proliferator-activated receptor γ: retinoid X receptor α heterodimer, thereby suppressing lipolysis-related genes [27,28].

Another adipocyte subpopulation was identified and characterized by abundant expression of *Xdh*, *Gda*, *Uap1*, and *Samhd1* (Fig. S5F and Table S6). Since these genes are involved in nucleotide metabolism, we annotated this subpopulation as the nucleotide metabolic adipocyte (NMA). The adipocyte subpopulation enriched a set of adhesion molecules, including *Cdh13*, *Adgrl4*, *Pkp4*, and *Adgrf5* (Fig. S5G and Table S6), and was annotated as the adhesive adipocyte (AA). The presence of AA was validated by single-molecule fluorescent in situ hybridization (smFISH) of its marker gene *Etl4* (Fig. 3E). Further, *Etl4*^+^ adipocytes were increased in number after cold exposure in mouse iWAT (Fig. 3E).

We annotated another adipocyte subpopulation as antigen-presenting adipocyte (APA) based on the abundant expression of genes involved in major histocompatibility complex class II (MHCII) antigen processing and presentation, including *Cd74*, *H2-eb1*, *Lgmn*, and the major costimulatory gene *Cd86* (Fig. S6 and Table S6). This expression characteristic suggests that APA likely carries out MHCII antigen presentation, the presence of which has been experimentally demonstrated [29]. Consistent with this conclusion, all the enriched TFs in the APA subpopulation are related to inflammatory responses, including *Bach1*, *Satb2*, *Rel*, and *Nfkb1* (Fig. 3D).

Following cold exposure, an additional subcluster characteristic of the beige adipocyte appeared in iWAT, which was high in beige marker genes, including *Ucp1*, *Esrrg*, *Gk*, *Ppargc1a*, and *Elov6* (Fig. 3F and Table S6). ProFAT [30], a tool to quantify the thermogenic potential of the adipocytes, derived a browning probability coefficient of >0.99 for beige adipocytes that is the highest among all the adipocyte subclusters (Fig. 3G). This result is consistent with our RNA velocity analysis that beige adipocytes are likely converted from LGAs and LSAs. Interestingly, although we did not detect a *Cyp2e1*^+^ adipocyte subcluster in iWAT as found in BAT by Sun et al. [15], expression of its major marker genes (such as *Cyp2e1*, *Atp4b4*, and *Aldh1a1*) was absent in beige adipocytes (Fig. S7). This observation suggests that the acetate-induced adipocyte whitening pathway is suppressed during beiging in an autonomous manner.

### The APA subpopulation is differentiated from CD74^+^ ASPC and increased by cold

**Fig. 4.**
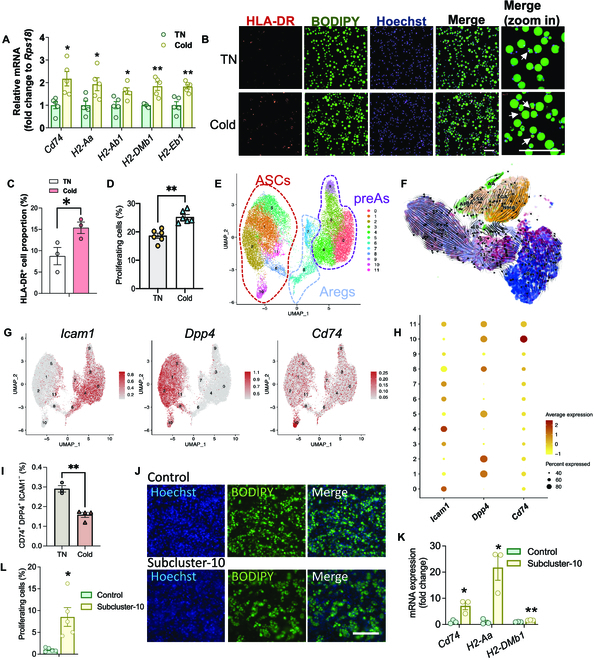
MHCII^+^ adipocytes are functionally increased in cold-adapted iWAT. Eight-week-old male C57BL/6J mice were either housed at TN (30 °C) or put to cold temperature (18 °C for 14 days followed by 6 °C for 7 days). The mature adipocytes were isolated from the iWAT for further analysis. (A) mRNA expression of MHC-II associated genes and (B) immunofluorescence staining of HLA-DR in mature adipocytes. Scale bars, 200 μm. (C) Quantification of (B). (D) MLR assay to quantify the T cell proliferation in cocultures. (E) Subclustering of ASPCs. (F) RNA velocity of ASPCs and adipocyte subclusters. (G) Feature plots of *Dpp4*, *Icam1*, and *Cd74* in ASPC subclusters. (H) Bubble chart of ASPC subclusters. (I) Percentage of CD74^+^ ASC in iWAT by flow cytometry. (J and K) Subcluster-10 and the rest of the ASPCs (control) were sorted from iWAT SVF and differentiated in vitro. (J) Adipogenesis was assessed by BODIPY staining. Scale bar, 200μm. (K) Relative mRNA expression of MHCII-related genes. (L) MLR assay to quantify the T cell proliferation in cocultures. *P < 0.05 and **P < 0.01.

We found a 2.5-fold increase in the APA subpopulation following exposure to cold (Fig. 3H and I). Indeed, the mRNA expressions of those genes associated with MHCII antigen presentation activity were uniformly up-regulated in the adipocytes after cold exposure (Fig. 4A). Immunofluorescence staining of HLA-DR, an MHC class II cell surface receptor, showed that the percentage of HLA-DR^+^ adipocytes was elevated after cold exposure (Fig. 4B and C). Pertinent to these observations, the mixed lymphocyte reaction (MLR) assay, which measures the antigen presentation activity of the cocultured cells, showed that T cells of the BALB/c background displayed greater alloresponsiveness when coincubated with the adipocytes from mice housed under cold condition (Fig. 4D). These results demonstrate that the MHCII antigen presentation activity of the adipocytes is potentiated after cold exposure.

To identify the APA precursor cell, we resubclustered ASPCs by increasing the resolution and obtained 13 ASPC subclusters (Fig. 4E). RNA velocity analysis showed that the APA subpopulation is most likely derived from ASPC subcluster-10 (Fig. 4F). Consistently, similar to APA, ASPC subcluster-10 is also high in *Cd74* expression (Fig. 4G and H). The percentage of ASPC subcluster-10 was counted by flow cytometry [CD45^−^, DPP4^+^, intercellular adhesion molecule-1-negative (ICAM1^−^), and CD74^+^] and was significantly decreased following cold exposure (Fig. 4I). To further interrogate the adipogenic function of this subcluster, subcluster-10 was sorted and differentiated in vitro, and, as shown by the BODIPY staining, it was readily differentiated into lipid-laden adipocytes (Fig. 4J). Furthermore, the adipocytes derived from subcluster-10 exhibited higher expression levels of genes associated with MHCII antigen presentation, compared to the adipocytes derived from the rest of the ASPC subpopulations (Fig. 4K). More importantly, the MHCII antigen presentation activity of the adipocytes differentiated from subcluster-10 was higher compared to those from the rest of the SVCs, as measured by the MLR assay (Fig. 4L). The results thus demonstrate that these CD74^+^ ASPCs are likely the precursor cells of the APA.

### Beige adipocytes are derived from LGAs

**Fig. 5.**
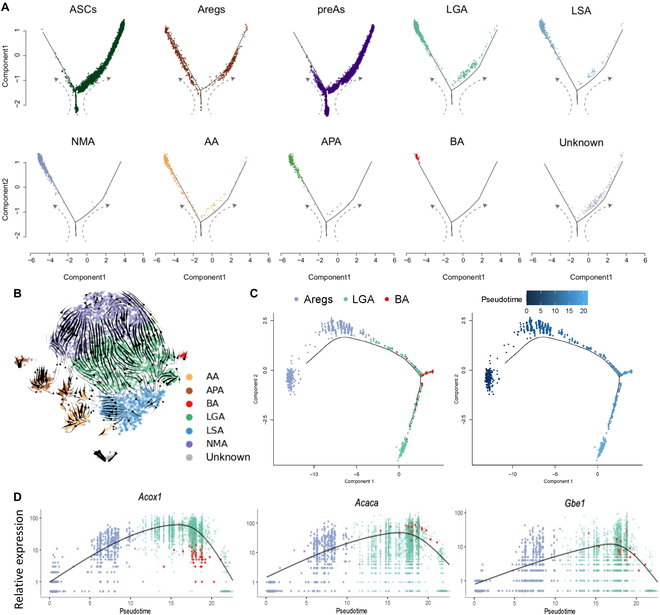
Beige adipocytes are transdifferentiated from LGAs. (A) The trajectory inference of ASPC and adipocytes subpopulations. (B) The RNA velocity inference of adipocyte subpopulations. (C) The trajectory inference of Areg cells, LGA, and beige adipocyte. (D) Rolling-wave plot showing the spline-smoothed expression patterns of significant pseudotime-dependent genes ordered according to pseudotime point of peak expression.

Beige cells have been suggested to arise from de novo differentiation from resident precursors or through reprogramming of existing mature adipocytes [19,31,32]. We interrogated our database that sequenced both ASPCs and mature adipocytes and sought to trace the origin of beige cells by mapping the developmental trajectory of these subpopulations (Fig. 5A). Among the 3 ASPC subpopulations, beige adipocytes are most likely derived from Areg cell, but not from ASC and preA (Fig. 5A). Since the trajectory analysis cannot clearly differentiate the developmental trajectory among adipocyte subclusters, RNA velocity analysis was conducted to interrogate the intercellular conversion among adipocyte subclusters. The result showed that beige adipocytes were derived from LGAs (Fig. 5B). Therefore we performed trajectory among Areg cells, LGAs, and beige adipocytes, which revealed a sequential developmental trajectory from Areg cells to LGAs, followed by conversion to beige adipocytes (Fig. 5C). By analyzing the pseudotime-dependent genes, genes including *Acox1*, *Acaca*, and *Gbe1*, which are respectively key genes in very-long-chain fatty acid oxidation, de novo lipogenesis, and glycogen synthesis were up-regulated during conversion to beige adipocytes (Fig. 5D), indicating that metabolism of very-long-chain fatty acid and glycogen is induced and involved in beige cell biogenesis.

### Cold exposure enhances the biogenesis of Bank1^+^ and Skap1^+^ endothelial cells

**Fig. 6.**
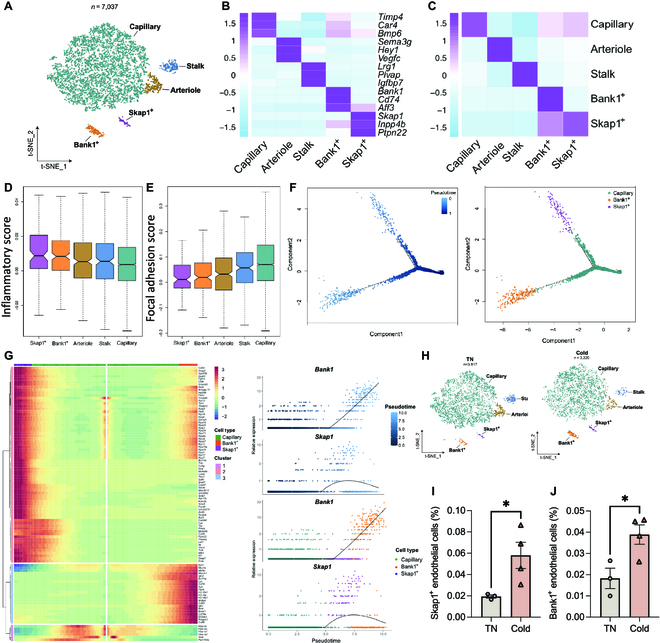
Endothelial cells are derived from stalk cells in iWAT. (A) t-SNE of endothelium subpopulations. (B) Heatmap showing scaled average expression of selected cell-type-enriched marker genes. (C) Heatmap showing average scaled gene module scores for the top 50 most enriched expressed marker genes in each cluster. (D and E) Inflammatory score and focal adhesion score of endothelium subpopulations. (F) The trajectory inference of capillary cells and Bank1^+^ and Skap1^+^ endothelial subclusters. (G) Rolling-wave plot showing the spline-smoothed expression patterns of significant pseudotime-dependent genes ordered according to pseudotime point of peak expression. (H) t-SNE of endothelium subpopulations in iWAT of TN and cold mice. (I and J) Flow cytometry analysis of Skap1^+^ and Bank1^+^ endothelial cells in mouse iWAT at TN or after cold. *P < 0.05.

Reclustering of endothelial cells (*n* = 7,037) resulted in 5 distinct subpopulations (Fig. 6A). We annotated 3 of these clusters as capillary cells (*Timp4*^+^), arteriole cells (*Sema3g*^+^), and stalk cells (*Fxyd5*^+^) based on the highly variable genes of each cluster and classical marker genes (Fig. 6B and C, Fig. S8A, and Table S7). Capillary cells comprise the most abundant endothelial cells in iWAT (Fig. 6A), which is in accordance with an scRNA-seq study of endothelial cells in other tissues [33]. Arteriole cells are the second abundant endothelial cells in iWAT. Stalk cells are a subgroup of endothelial cells that are activated during angiogenic sprouting [34]. In addition, we discovered another 2 previously unreported endothelial subpopulations, which were annotated as Bank1^+^ and Skap1^+^ endothelial cells by their most distinctive marker genes, respectively (Fig. 6B and C, Fig. S8A, and Table S7). We further characterized the genes differentially expressed across the conversion process in Bank1^+^ and Skap1^+^ cells, and the top enriched pathways are related to immune responses (Fig. S8B and C). Kyoto Encyclopedia of Genes and Genomes analysis revealed that Bank1^+^ cells are enriched for B cell receptor signaling, while genes highly expressed in Skap1^+^ cells are enriched for T cell receptor signaling and ICOS-ICOSL signaling in T helper cells (Fig. S8B and C). We compared the inflammatory and focal adhesion and monocyte adhesion scores among all of the endothelial subpopulations. *Bank1^+^* and *Skap1^+^* cells show the highest inflammatory scores, while the monocyte adhesion scores were relatively low among all the subpopulations (Fig. 6D and Fig. S8D). They also had the lowest focal adhesion score (Fig. 6E), suggesting that these 2 subpopulations are not directly involved in leukocyte adhesion but play a role in vascular structural remodeling. Pseudotime trajectory analysis indicated that Bank1^+^ and Skap1^+^ endothelial cells derive from capillary cells (Fig. S8E). To further interrogate this hypothesis, we performed cell trajectory inference among these 3 clusters, which predicted that the capillary cells transit toward Bank1^+^ and Skap1^+^ cells (Fig. 6F). Among all the pseudotime-dependent genes, *Bank1* and *Skap1* genes were enriched toward the end of the 2 cell clusters, respectively (Fig. 6G and Table S8).

Comparison of the composition of endothelial cell subpopulations showed the relative number of stalk cells decreased and the relative number of Bank1^+^ and Skap1^+^ cells increased following cold exposure compared with the thermoneutral condition (Fig. 6H and Fig. S8F). The increase in Bank1^+^ endothelial cells, which is high in CD74 expression, and Skap1^+^ endothelial cells in iWAT undergoing cold stimulation were validated by flow cytometry (Fig. 6I and J and Fig. S8G). These observations provide additional evidence that the biogenesis of the immune-competent endothelial cells in iWAT is potentiated by cold exposure.

### Adipose Schwann cells are remodeled by cold exposure

In Schwann cells a number of genes were modulated by cold (Fig. S9 and Table S9). The top 2 up-regulated genes *Amphiregulin* (*Areg*) and *Atf3* are implicated in Schwann cell growth and axonal outgrowth [35,36], demonstrating that cold exposure vividly induces peripheral nerve growth. Interestingly, the most 2 down-regulated genes in Schwann cells encode proteins for tryptophan metabolism (*Ido1)* and sodium potassium transport (FXYD domain containing ion transport regulator 2, *Fxyd2*), respectively, both of which are highly relevant to the function of Schwann cells. These findings suggest that there is profound remodeling in Schwann cells after cold exposure.

### Adipose SMCs are remodeled by cold exposure

In SMCs, among the top 5 most up-regulated genes, 3 of them, i.e., *Acaca*, *Acsl1*, and *Scd1*, were involved in lipid metabolism (Fig. S9B and Table S10), suggesting that the energy metabolism, at least the lipid metabolism, was substantially altered by cold exposure.

### Integrative analysis of immune, nonimmune cells, and adipocytes before and after cold exposure

To investigate the possible cell–cell cross-talk between the immune cells with the other cell types in our dataset, we integrated the single-cell sequencing (sc-seq) dataset including the total stromal vascular cells in mouse iWAT with or without cold exposure [37] with ours for analysis (Fig. S10A and B). No obvious changes in cellularity were found under different housing conditions (Fig. S10C). Nevertheless, it is interesting to note that the intercellular communications between the immune cells with other cells were altering in response to cold (Fig. S10D to F and Table S11). The cellular interactions were generally enhanced between most of the cells after cold exposure (Fig. S10F). In particular, the input signals from the ASPCs, adipocytes, and SMCs to CD4^+^ and CD8^+^ T cells were potentiated under cold condition, and the outgoing signals from SMCs to B cells were found increased as well (Fig. S10F). In contrast, the monocytes received less incoming signal strength especially from the ASPCs and Schwann cells (Fig. S10F).

## Discussion

Cold exposure remains the most prominent stimulus to evoke beiging in WAT [20,32,38]. Intriguingly, mild but chronic cold exposure is showing promise to counteract obesity, diabetes, atherosclerosis, and even cancer [39–42]. However, cold exposure is clinically impractical, because of the inconvenience of the regimen, the unfavorable feeling, as well as the complex and indirect pathways elicited by cold. Although it is understood that cold exposure induces the biogenesis and mobilization of beige adipocytes, whether and how the status of white adipocytes are altered in this process is still unclear. Furthermore, it remains unexplored the responsiveness to chronic cold exposure of other nonimmune cell types present in the iWAT, such as ASPCs, endothelial cells, SMCs, and Schwann cells. Thus, the cellular and molecular events at the single-cell resolution will be informative to address these questions. Geared toward this, we applied snRNA-seq and scRNA-seq to characterize the transcriptome of iWAT from mice of both sexes with or without chronic cold stress. This allowed us to gain a comprehensive view of the cellular and intercellular events within WAT at thermoneutrality and in the context of cold adaptation. The dataset obtained also serves as a public repository to unravel the sophisticated cellular mechanisms underlying white adipose physiology.

Recent sn-seq studies have demonstrated the heterogeneity of mature white adipocytes as manifested by the presence of distinct adipocyte “subpopulations” [17,18,22]. Sárvári et al. [17] found LGA, LSA, and stressed lipid scavenging adipocytes in mouse epididymal WAT, the former 2 of which are also identified in iWAT in our study. In addition to LGA and LSA, we found another 4 subpopulations in iWAT at thermoneutrality. The NMA subpopulation is abundant in genes encoding nucleotide metabolism enzymes, such as xanthine dehydrogenase, which oxidizes the purine molecule xanthine to uric acid. Interestingly, a recent study reported that apoptotic brown adipocytes release a specific pattern of metabolites enriched in purine metabolites, which enhances the thermogenic programme in healthy brown adipocytes [43]. Although it is yet to be examined whether purine stimulates the cyclic adenosine monophosphate-activated protein kinase A signaling pathway in white and beige adipocytes as in brown adipocytes, it will be interesting to interrogate whether the NMA subpopulation serves as a purine sink to modulate adipose biology.

APA, which is characterized by expression of MHCII antigen presentation-related genes, displays transcription profiles less relevant to the existing adipocyte subclusters, suggestive of its distinct developmental lineages from the other adipocyte subpopulations. Indeed, we provide experimental evidence that the APA subpopulation is likely differentiated from a subcluster of ASPC with high CD74 expression. Notably, a decrease in ASPC and an increase in the APA subpopulation were observed in iWAT after cold exposure. Lineage tracing experiment will also be warranted in future studies to validate this trajectory in vivo. Characterization of the precursor cell of APA will substantially foster the further interrogation on the function of APA.

Work by Deng et al. [29] demonstrated that in obese visceral adipose tissue of both mouse and human, adipocyte MHCII activity was potentiated and directly activated by CD4^+^ T cells, leading to aggravated adipose inflammation. In particular, genetic deficiency of adipocyte MHCII decreases adipose interferon-γ expression and increases adipose regulatory T cell (T_reg_) abundance in epididymal WAT. Thus, an adipose T cell subset switch is responsible for decreased adipose inflammation and improved insulin resistance in obesity. Resident T_reg_ in eWAT is much more abundant in number compared to iWAT and expresses a distinct T cell receptor repertoire compared to other T_regs_ in the lymph node [44,45]. The function of the APA subpopulation in iWAT remodeling and whether it causes an adipose T cell subset switch in a similar way as in eWAT upon cold challenge will be explored using the adipocyte-selective MHCII knockout mice. In addition, the alloantigen presented by APAs under cold stimulation will be elucidated in future studies.

The source of the beige adipocyte has been a topic of high debate. It may arise from white adipocytes via transdifferentiation in a reversable manner based on electron microscopy and pulse-chase lineage-tracing studies [32,46,47]. Alternatively, other studies found that committed beige precursors are present and reside in the adipose tissue vasculature [31,48,49]. Discrepant findings from various studies may be confounded by the different methods and animal models used, such as the utilization of different Rosa26 reporter, the slight genetic variance in mouse strains used by different laboratories, and the methods used to visualize reporter expression. Our data suggest a combined mechanism involving both transdifferentiation and de novo adipogenesis, in that Areg cells differentiate to LGAs, followed by transdifferentiation to beige adipocytes. This might also explain the seemingly contradictory conclusions among different studies.

The 2 disseminating work to carry out the scRNA-seq study in adipose stromal cells identified the Areg cell as a subpopulation of ASPC, which is characterized by high CD142 expression [9,10]. In vivo transplantation study showed that ASC (DPP4^+^ progenitors) gives rise to Areg in vivo [9]. Upon cold exposure, the comparative decline in ASC abundance was associated with an increase in Areg abundance, pointing to a possibility that cold stimulus enhances the conversion from ASC to Areg. This mechanism is consistent with a high plasticity in Areg cell as uncovered by our study that several metabolic genes were up-regulated after cold exposure. However, there is an inconsistency concerning the adipogenesis of Areg cell in that Schwalie et al. [10] found that Areg cells were refractory to adipogenesis, while work from Seale’s group [9] showed comparable adipogenic capacity between Aregs and preAs. A similar subpopulation is also present in eWAT [17]. Just before the submission of this manuscript, Zachara et al. [50] revealed that Areg cells exhibit temporal phenotypic alterations that can be both non- and antiadipogenic in an age-dependent manner. The inhibitory nature of Areg is driven by specific secretory factors that cooperate with the retinoic acid signaling pathway. Thus, our findings and others imply that Areg cell may represent a plastic and versatile ASPC subpopulation, and the discrepancies in adipogenic potential are possibly due to the dynamic status of Aregs and the distinct adipose tissue microenvironment in mice of different age and conditions. Given the plastic nature of Areg cells, it is also possible that under appropriate conditions (such as age, ambient temperature, and adipose depot microenvironment), the biology of Aregs might be different, and they might differentiate directly to beige adipocytes as well. Therefore, it remains an intriguing question whether cold stimulation alters the intrinsic features of Aregs or the adipose niche, consequently contributing to the biogenesis of beige cells from Areg cells.

Despite multivariant changes in cell subtype changes, we did not see obvious changes in iWAT cellularity, relative to the dramatic changes in cellular status. This coincides with the fact that the cellular development (hypercellularity) in adipose tissue is mainly found in early onset of obesity or under extreme obese condition and stays constant in adulthood [51,52]. It is also worth noting that in a recent comprehensive sn-seq analysis of adipose tissue between lean and obese individuals, similar conclusion is obtained that little cellularity is observed between different groups of people [18]. In line with this notion, under mild and chronic cold exposure condition, cellularity is likely not a predominant mechanism for iWAT remodeling.

In the current study, thermoneutrality (30 °C), instead of room temperature, was used as the control condition since the room temperature (22 to 24 °C) is the thermoneutral temperature for human, while for mammals of smaller size including mice, room temperature is below their thermoneutral zone (29 to 34 °C). Therefore, at this condition, mice are believed to be at a mild chronic thermal stress and are suboptimal for interpreting the cold-stimulation-related observations.

In conclusion, we provide a comprehensive map of the nonimmune cells in mouse iWAT at single-cell resolution and provide a description of cellular remodeling from thermoneutrality to chronic cold exposure (Fig. S10). Recently, sn-seq of adipose tissue between lean and obese individuals was elegantly performed [17,18]. However sampling of human adipose tissue with cold exposure is more challenging. In this study, changes in cell subpopulation composition, gene expressions, secretory profiles, and intercellular cross-talk within mouse iWAT are disclosed. In particular, some previously unidentified adipocyte and endothelial subpopulations (or status) were discovered and showed functional relevance to cold-evoked WAT remodeling. Insights on the biogenesis of beige adipocytes, as well as adipocytes with antigen-presenting activity, were provided. Furthermore, the intercellular networking, including the cell-type-specific secretome and ligand–receptor pairs under thermoneutrality and cold conditions, was enlisted. All of these and those beyond our analysis in this study will be valuable source of information to spur future investigation into the adipose biology on a hypothesis-driven mode. The new subpopulations and cell states discovered in endothelial cells and mature adipocytes can be examined in future in clinical samples, and the implication in metabolic diseases can be explored.

### Limitations of study

To identify the general, gender-independent changes, both male and female mice were used in the current study, and the isolated cells were pooled before the library construction. Dataset and analysis of different genders certainly merit further investigation, to further reveal sex-specific molecular events. Second, immune cells were excluded from sampling as we were focusing on the adipogenic trajectory toward beige cell biogenesis. Delineation of the immune cell changes using sc-seq technology in response to cold exposure in future will add another layer to our understanding of adipose remodeling by cold. Nevertheless, we merged our in-house generated data with a previously published sc-seq dataset that includes the immune cells in mouse iWAT with or without cold stimulation [53]. Interestingly, differential cell–cell interactions between immune cells with other cell types were observed, which well deserve future investigation to interrogate how the immune cells contribute to cold-evoked adipose beiging. Of course, the bioinformatics findings are to be experimentally verified, especially because the experimental conditions between the 2 studies are not identical.

## Methods

### scRNA-seq and snRNA-seq data processing

The 10x raw data were each processed with Cell Ranger (version 4.0.0). Expression data were obtained from the cellranger count on the prebuilt mouse reference set (mm10). We used default parameters for quality control and produced for each sample a barcodes.tsv, genes.tsv, and matrix.mts file. These data were loaded into the Seurat v4 in R to perform standard procedures for filtering, normalization, and integration of cells using methods described previously. Briefly, cells with <20% mitochondrial DNA [50], >200 unique UMI counts, and <6,000 unique UMI counts based on their distribution in the sample were remained for further analysis. Counts were normalized to obtain correct relative gene expression abundances between cells [51]. Sn-seq and sc-seq data were integrated through the *FindIntegrationAnchors* and *IntegratedData* functions of the Seurat package, where the *FindIntegrationAnchors* function was used to identify anchors and the *IntegrateData* function was used the identified anchors to integrate the dataset [52].

Variable genes were selected by at least a 0.25 log fold change (FC) in gene expression between the groups. Variable genes (*N* = 2,000) were projected onto a low-dimensional subspace using principal components analysis. Cells were then clustered and visualized onto 2 dimensions using t-SNE or UMAP. Cell types were annotated by highly variable genes and known marker genes of each cluster.

### Subpopulation analysis

Cells previously annotated as ASPCs, endothelial cells, and adipocytes were subset and reclustered using methods described above. Cell subpopulations were identified by highly variable genes and known maker genes.

### Developmental trajectory inference

For differentiation trajectory analysis, Monocle (version 2.14.0) algorithm with the signature genes from *differentialGeneTest* function was used. The differentiation trajectory of selected cells was inferred with the default parameters. The pseudotime-related genes were calculated by *differentialGeneTest* function, and dot plots for the selected genes were generated using the Monocle function *plot_genes_in_pseudotime.* For RNA velocity analysis, scVelo was adopted with the default parameters. The count matrices were size-normalized to the median of total molecules across cells. The top 2,000 highly variable genes were selected for spliced and unspliced mRNA. For velocity estimation, first- and second-order moments were computed for each cell across its 30 nearest neighbors.

### DEG analysis

Differential gene expression analysis was conducted using DESeq2 in R (version 4.0.0), with the filtering threshold set at an FC of >1.5 and the false-discovery-rate-adjusted *P* < 0.05.

### Gene Ontology and Kyoto Encyclopedia of Genes and Genomes analysis

Gene Ontology and pathway analysis were performed using clusterProfiler in R (version 4.0.0) and QIAGEN Ingenuity Pathway Analysis.

### Secreted factor analysis

The secreted-related proteins were first downloaded from the UniProtKB database, and then classical and nonclassical secreted proteins were identified using singnalP 5.0 (SP value > 0.45) and SecretomeP 2.0 (NN-score > 0.6) to obtain the mouse secreted factor database (Table S12). The differentially expressed secreted factors in each cluster were then identified (FC > 1.5 or FC < 0.67, *P* < 0.05).

### Animals

Mice were housed at the environmental chamber (Dowsontec) with access to food and water ad libitum for different housing temperatures. For stepwise cold exposure, 6- to 8-week-old C57BL/6J mice were housed at thermoneutrality (30 °C) for 2 weeks, followed by housing at 18 °C for 2 weeks and subsequently 6 °C for 1 week. For thermoneutrality group, the mice were housed at 30 °C for 5 weeks. The measurement of basal metabolic rate was described in a previous paper [53]. Briefly, the mice were anesthetized by pentobarbital (90 mg/kg, intraperitoneally), after which the body oxygen consumption values were measured by putting the mice in metabolic cage (PromethION) for 45 min at 34 °C. All animal procedures were performed under the guidance of Department of Health, the Government of the Hong Kong Special Administrative Region, and the Chinese University of Hong Kong Laboratory Animal Service Centre (reference no. 21-051-MIS).

### Cell isolation

Dissected inguinal subcutaneous WAT was minced and digested in Dulbecco’s modified Eagle’s medium (DMEM; Gibco) with collagenase II (2 mg/ml; Gibco) and 3% bovine serum albumin (BSA) for 30 min at 37 °C in CO_2_ incubator without shaking. The digestion was filtered through a 100-μm cell strainer (Jet Biofil) and centrifuged at 600*g* for 10 min. The pelleted cells [stromal vascular fraction (SVF)] were collected for further staining or flow cytometry. The upper layers (adipocytes) were subjected to further staining, RNA purification, or nuclei isolation with nuclei and cytosol isolation kit for adipose tissue (Minute, #AN-029).

### Immunofluorescence staining

In regard of primary adipocyte staining, isolated primary adipocytes were fixed in fixation buffer [2% paraformaldehyde and 1% sucrose in phosphate-buffered saline (PBS)] for 15 min, followed by blocking in block buffer (10% mouse serum and 1% BSA in PBS) for 30 min. Blocked adipocytes were then successively incubated with primary antibody, HLA-DR (1:250; Thermo Fisher Scientific, #MA5-11966, LN3), for 1.5 h and secondary antibody, Alexa Fluor 568 goat anti-mouse immunoglobulin G (1:500; Thermo Fisher Scientific, #A11031), Hoechst 33342 (Thermo Fisher Scientific, #62249), and BODIPY FL C_12_ (Thermo Fisher Scientific, #D3822), for 30 min. Stained adipocytes were resuspended in 80% glycerol and mounted on slides with iSpacer (SunJin Lab, #IS007). Whole staining procedure was performed at room temperature under gentle rotation. Then, images were taken with LSM900 microscope (ZEISS).

For differentiated cells in 384-well plate, added induction medium [DMEM, 20% fetal bovine serum (FBS), 20 nM insulin, 1 μM dexamethasone, 0.5 μM isobutylmethylxanthine, and 1 μM rosiglitazone] within 48 h after cell seeding for 2 days. Then, maintenance medium (DMEM, 10% FBS, 20 nM insulin, and 1 μM rosiglitazone) was replaced every 2 days for 6 days. Fully differentiated adipocytes were incubated with Hoechst 33342 and BODIPY FL C_12_ for 30 min before imaging with Ti2-E microscope (Nikon).

### Isolation of CD45^−^ stromal vascular cells and adipocyte nuclei for sc-seq and sn-seq

SVF cells from subcutaneous adipose tissue were resuspended in Hanks’ balanced salt solution buffer for incubation with CD45 antibody (1:200; BioLegend, #25-0451-82, 30-F11) for 30 min at 4 °C. 7-Aminoactinomycin D (7-AAD; 1:1,000; BioLegend, #420404) was added 10 min before fluorescence-activated cell sorting (FACS). The cells were sorted with MoFlo XDP sorter (Beckman Coulter) with a 100-μm nozzle. For isolation of primary adipocytes nuclei, purified nuclei from adipocytes were resuspended in PBS supplemented with recombinant ribonuclease inhibitor (0.5 U/μl; Takara, #2313B). 7-AAD (1:1,000) was added 10 min before FACS. The nuclei were sorted with MoFlo XDP sorter (Beckman Coulter) with a 100-μm nozzle.

### MLR assay

Mouse spleen from BALB/c mice was minced and filtered through a 100-μm cell strainer in RPMI 1640 medium (Gibco), followed by red blood cell lysis with ammonium–chloride–potassium lysing buffer (150 mM ammonium chloride, 10 mM potassium bicarbonate, and 0.1 mM disodium EDTA). Mouse spleen cells were incubated with 5 μM carboxyfluorescein succinimidyl ester (BioLegend, #423801) in PBS for 30 min at 37 °C in water batch and then cocultured with isolated primary adipocytes from mouse iWAT in mixed medium (45% DMEM, 45% RPMI 1640, and 10% FBS) in 24-well plates with shaking at 60 rpm at 37 °C in CO_2_ incubator (adipocytes:spleen cells = 1:5). Three days after incubation, cells were resuspended in PBS and filtered through 40-μm strainers. 7-AAD (1:1,000) was added 10 min before flow cytometry in a FACSymphony SORP flow cell analyzer (BD Biosciences).

### Flow cytometry

SVF cells were resuspended in PBS with 3% BSA after TruStain FcX PLUS antibody (1:100; BioLegend, #156604, S17011E) blocking for 30 min for incubation with the following antibodies for 30 min at 4 °C: CD144-Alexa Fluor 647 (1:50; BD Biosciences, #562242, 11D4.1 ), CD74-Alexa Fluor 488 (1:100; BioLegend, #151006, In1/CD74), CD26 (1:100; DPP4)-phycoerythrin (BioLegend, #137803, H194-112), CD54 (ICAM1)-Alexa Fluor 647 (1:100; BioLegend, #116114, YN1/1.74), and CD45-Pacific Blue (1:100; BioLegend, #157212, S18009F). After extracellular staining, cells were fixed with 3% paraformaldehyde in PBS for intracellular staining. Cells were permeabilized with fixation/permeabilization kit (BD Biosciences, #554714), followed by incubation successively with primary antibody, SKAP55 (1:750; Abcam, #ab171947, EPR11359), and secondary antibody, anti-rabbit-Alexa Fluor 568 (1:600; Thermo Fisher Scientific, #A11036), for 45 min at 4 °C. Stained cells were analyzed in a FACSymphony SORP flow cell analyzer. For isolation of subcluster-10, SVF cells were resuspended in Hanks’ balanced salt solution with 3% BSA and 25 mM glucose after ammonium–chloride–potassium lysis for incubation with the following antibodies for 45 min at 4 °C: CD74-fluorescein isothiocyanate (1:100) and CD45-Pacific Blue (1:500). Stained cells were sorted in a FACSAria Fusion cell sorter (BD Biosciences). Sorted cells were seeded in 384-well plates in DMEM with 20% FBS.

### Single-molecule fluorescent in situ hybridization

smFISH was conducted on paraffin iWAT sections following the recently published SABER784 FISH protocol with minor modifications [53]. Gene-specific probe sets, branch probes, and the CY3-coupled signal probe were purchased from Servicebio. Briefly, the sections were subjected to dewaxing, dehydration, and retrieval with dewaxing transparent liquid (Servicebio), ethanol, and retrieval buffer (Servicebio). Tissue sections were then digested with proteinase K (20 μg/ml), followed by hybridization with 500 nM *Etl4* probe mixture (5′-CTGATACTCTGTCAAGTTTCCTTCGCT-3′, 5′-GGCAGAGGTTACTTCCTGTGTGATT-3′, CCGTTATAGTGTTCCAGATTTTGCC-3′, 5′-CCTGATCTCGTGGTGGTAAGTCCTG, and AACTGACCTTAGGCACATTTTTCTGG-3′), branching probe, and signal probe coupled with CY3. After nuclei staining with 4′,6-diamidino-2-phenylindole, images were taken with Nikon Eclipse Ci and then analyzed with Nikon DS-U3 and ImageJ.

### Real-time polymerase chain reaction

Total RNA was extracted by RNAiso Plus (Takara) and reverse-transcribed into cDNA using the PrimeScript RT Reagent Kit (Takara). Real-time polymerase chain reaction reactions were performed using SYBR Premix Ex Taq II (Takara) on a 7900HT (Applied Biosystems), with the ribosomal protein S18 (*Rps18*) gene as a normalization control. Primer sequences are listed in Table S13.

## Data Availability

All the data supporting the findings described in this manuscript are available in the article and in the Supplementary Materials.

## Supplementary Materials

Supplementary 1 Tables S1 to S13 Fig. S1. Gating strategy for isolation of CD45- stromal vascular cells and mature adipocyte nuclei. Fig. S2. Beiging of iWAT from mice undergoing stepwise cold adaptation. Fig. S3. Integration and clustering of single cell and single nuclei from mouse iWAT. Fig. S4. Characterization of ASPCs in iWAT from TN and cold adapted mice. (A) Feature plots of marker genes in ASPCs. (B)-(C) Volcano plot of DEGs in preAs (B) and ASCs (C). Fig. S5. Characterization of 7 adipocyte subclusters in mouse iWAT. Fig. S6 MHCII-active adipocytes are present in mouse iWAT. Fig. S7. Marker genes for Cyp2e+ cells that negatively regulate brown adipocytes were excluded in beige adipocytes. Fig. S8. Characterization of endothelial cells in iWAT from TN and cold adapted mice. Fig. S9. Gene expression was altered in iWAT schwann cells and SMCs by cold. Fig. S10. Integrative analysis of immune, non-immune cells and adipocytes in iWAT before and after cold exposure. Fig. S11. A single cell atlas consisting mature adipocytes and non-immune stromal vascular cells in mouse iWAT under thermoneutrality and after cold adaptation is depicted.
