# Supplementary material for: Cold-Induced Reprogramming of Subcutaneous White Adipose Tissue Assessed by Single-Cell and Single-Nucleus RNA Sequencing
Source: Research (Wash D C). 2023 Jun 28;6:0182. doi: 10.34133/research.0182 (PMC10308956; doi:10.34133/research.0182)
Supplement: Supplementary 1 — Tables S1 to S13 Fig. S1. Gating strategy for isolation of CD45- stromal vascular cells and mature adipocyte nuclei. Fig. S2. Beiging of iWAT from mice undergoing stepwise cold adaptation. Fig. S3. Integration and clustering of single cell and single nuclei from mouse iWAT. Fig. S4. Characterization of ASPCs in iWAT from TN and cold adapted mice. (A) Feature plots of marker genes in ASPCs. (B)-(C) Volcano plot of DEGs in preAs (B) and ASCs (C). Fig. S5. Characterization of 7 adipocyte subclusters in mouse iWAT. Fig. S6 MHCII-active adipocytes are present in mouse iWAT. Fig. S7. Marker genes for Cyp2e+ cells that negatively regulate brown adipocytes were excluded in beige adipocytes. Fig. S8. Characterization of endothelial cells in iWAT from TN and cold adapted mice. Fig. S9. Gene expression was altered in iWAT schwann cells and SMCs by cold. Fig. S10. Integrative analysis of immune, non-immune cells and adipocytes in iWAT before and after cold exposure. Fig. S11. A single cell atlas consisting mature adipocytes and non-immune stromal vascular cells in mouse iWAT under thermoneutrality and after cold adaptation is depicted. [file research.0182.f1.zip › scSeq_Supplemental figures_R1.pdf]

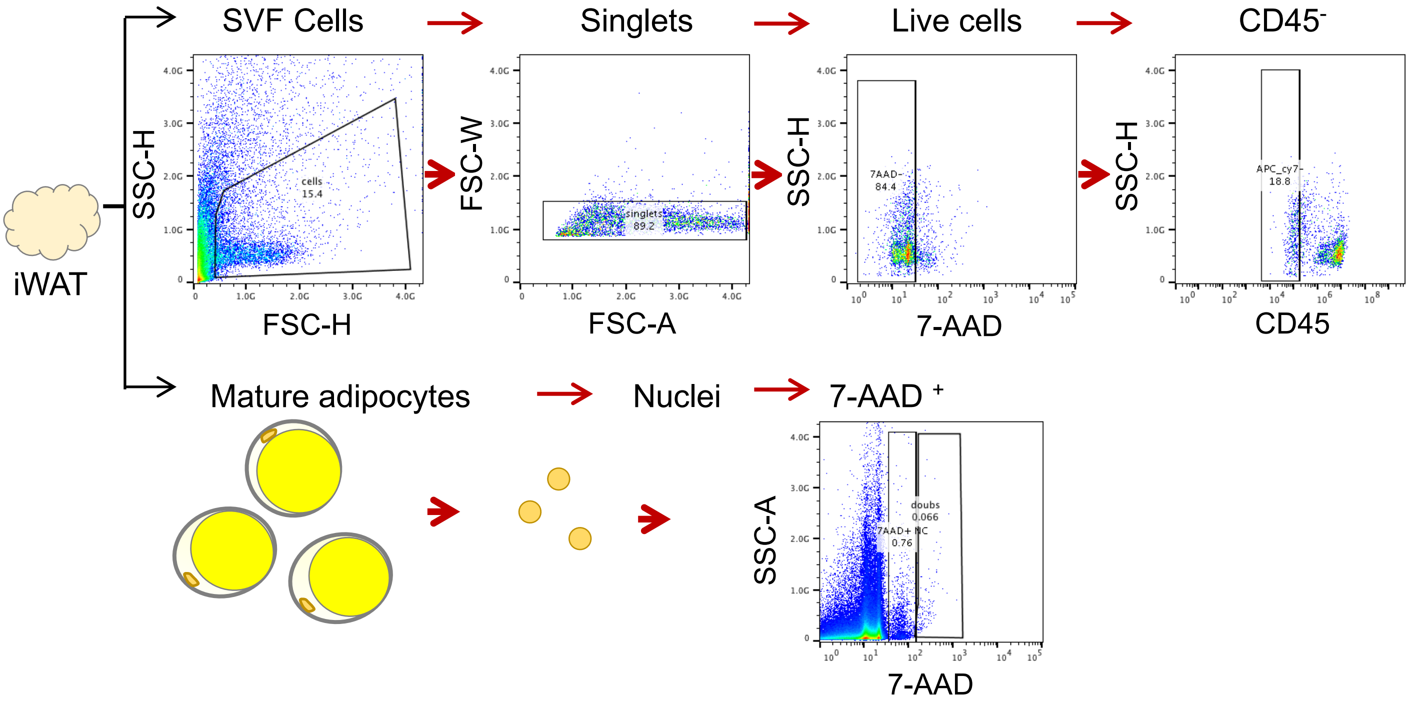

Figure S1. Gating strategy for isolation of CD45- stromal vascular cells and mature adipocyte nuclei.

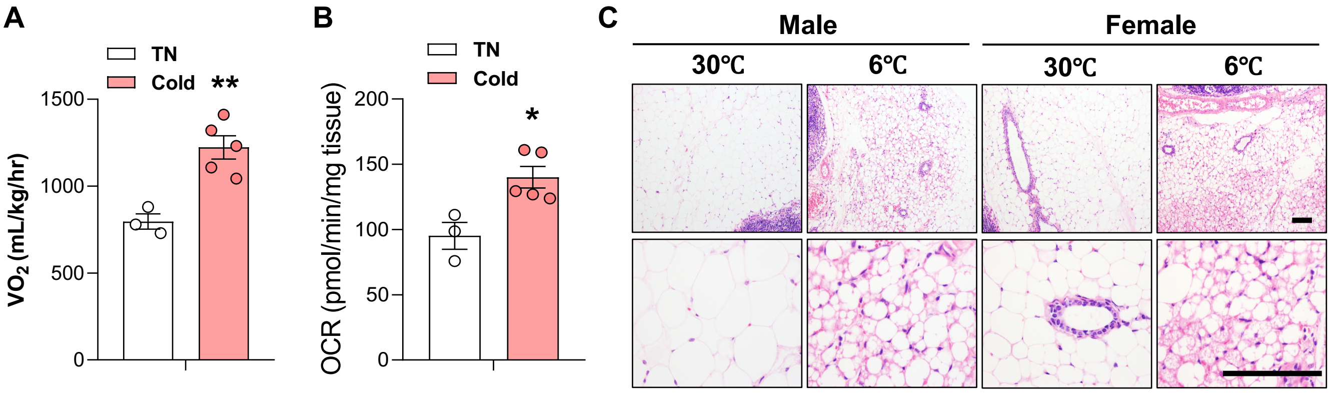

Figure S2. Beiging of inguinal subcutaneous white adipose tissue (iWAT) from mice undergoing stepwise cold adaptation. (A) Basal oxygen consumption of male mice. (B) Oxygen consumption rate of the iWAT dissected from male mice. (C) Representative images of the HE staining of the iWAT. scale bar=100μm

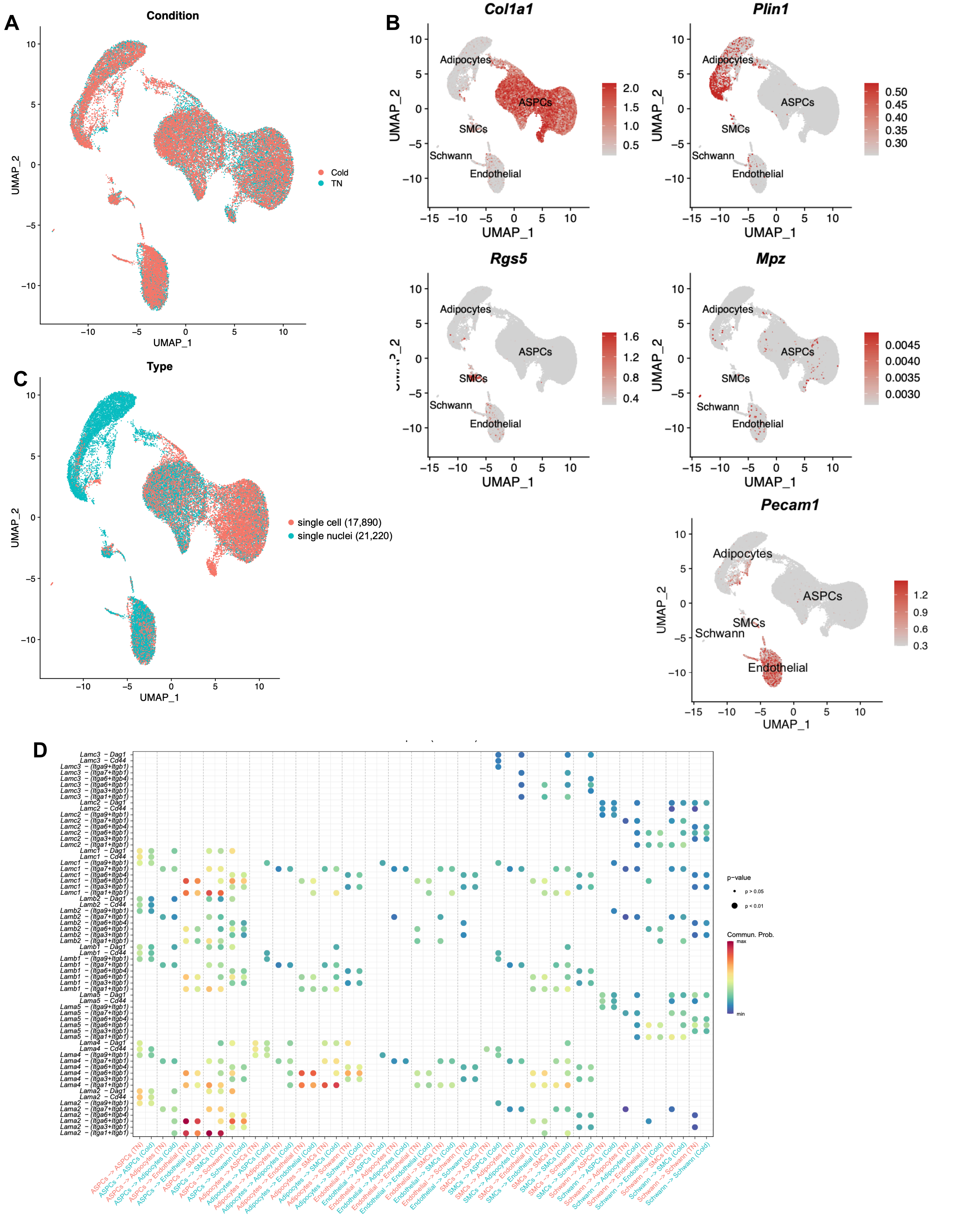

Figure S3. Integration and clustering of single cell and single nuclei from mouse inguinal subcutaneous white adipose tissue (iWAT). (A) UMAP of the cells grouped by cold and TN conditions. (B) UMAP of iWAT cells grouped by single cell and single nuclei. (C) Feature plots of marker genes of each cell type. (D) Laminin-related cell-cell communications among different cell types in TN and cold.

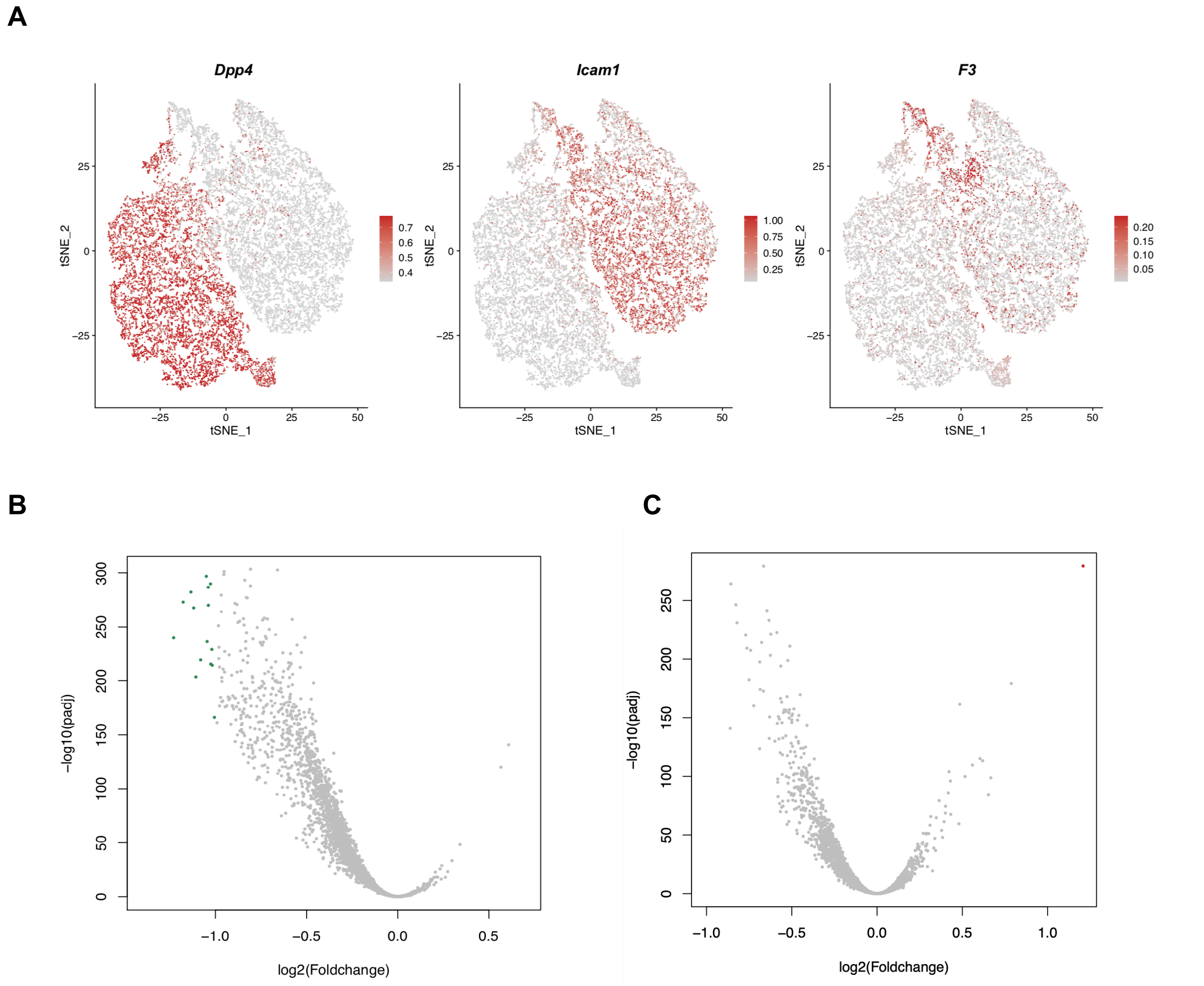

Figure S4. Characterization of ASPCs in iWAT from TN and cold adapted mice. (A) Feature plots of marker genes in ASPCs. (B)-(C) Volcano plot of DEGs in preAs (B) and ASCs (C).

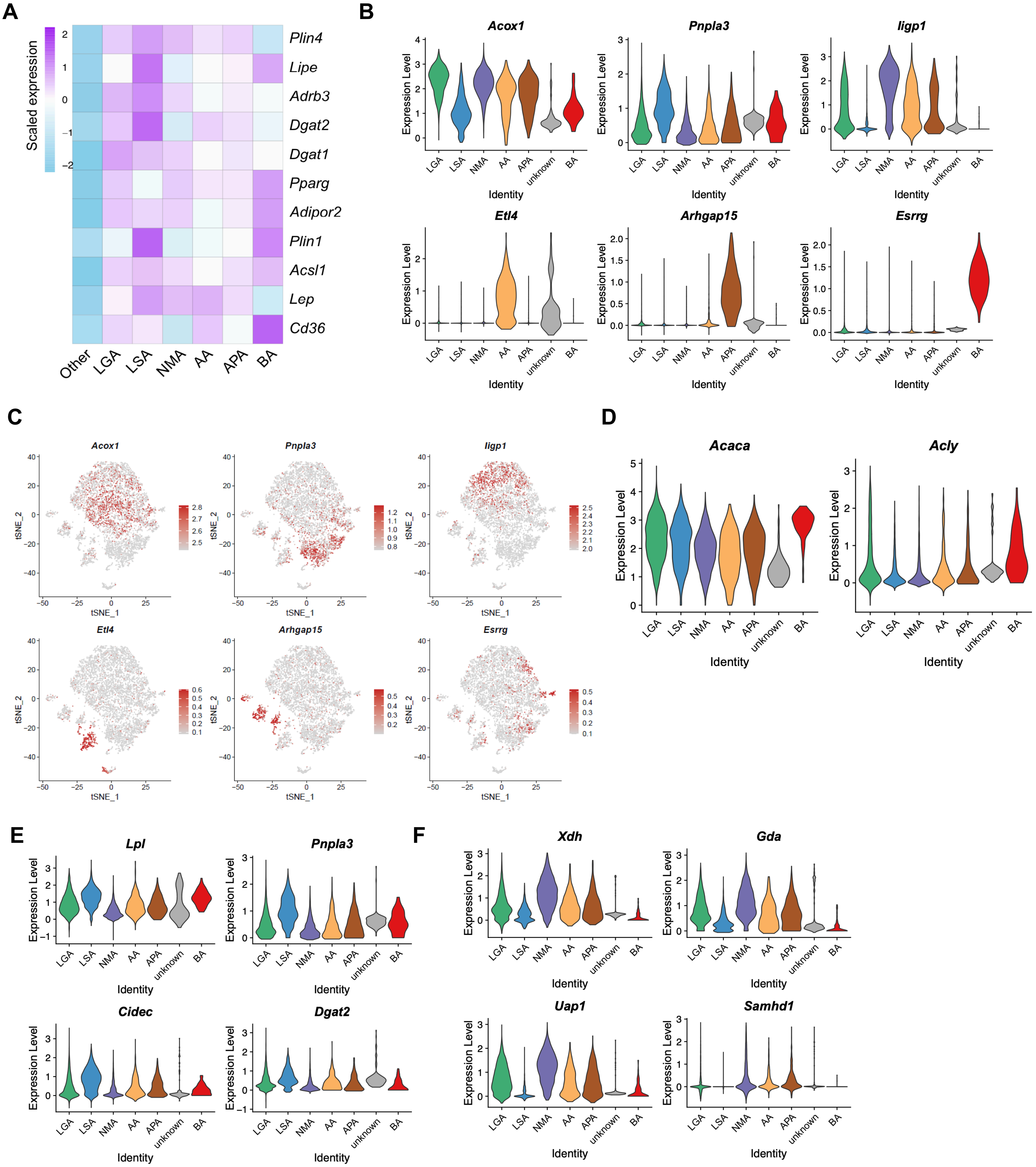

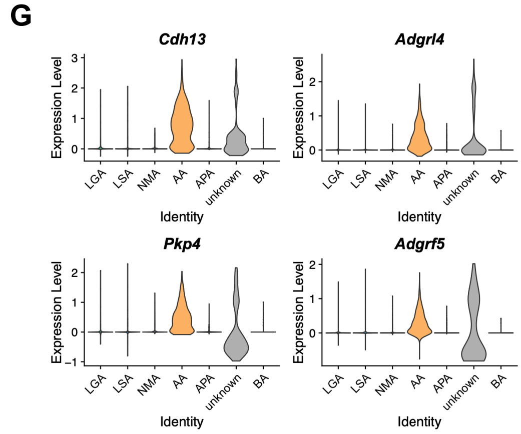

Figure S5. Characterization of 7 adipocyte subclusters in mouse iWAT. (A) Heatmap showing scaled average expression of a set of adipocyte marker genes across adipocyte subpopulations and in all non-adipocytes (others). (B) Violin plots and (C) Feature plots of marker genes in adipocyte subclusters. (D)-(G) Violin plots of genes involved in lipid biosynthesis (D), lipid uptake, transport and esterification (E), nucleotide metabolism (F) and cell adhesion (G).

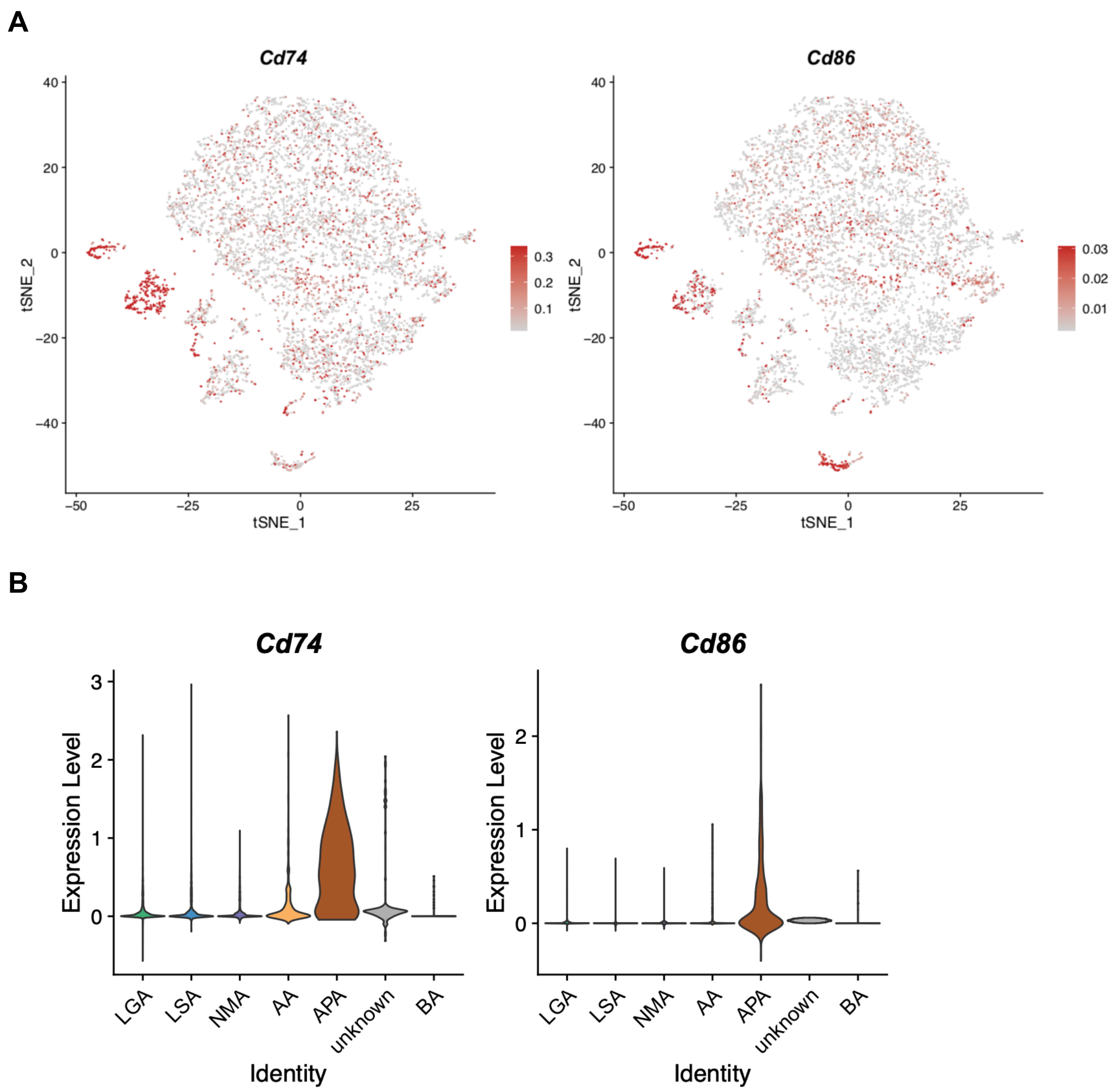

Figure S6. MHCII-active adipocytes are present in mouse iWAT. (A) Feature plots of *Cd74* and *Cd86* in adipocyte subclusters. (B) Violin plots of *Cd74* and *Cd86* in adipocyte subclusters.

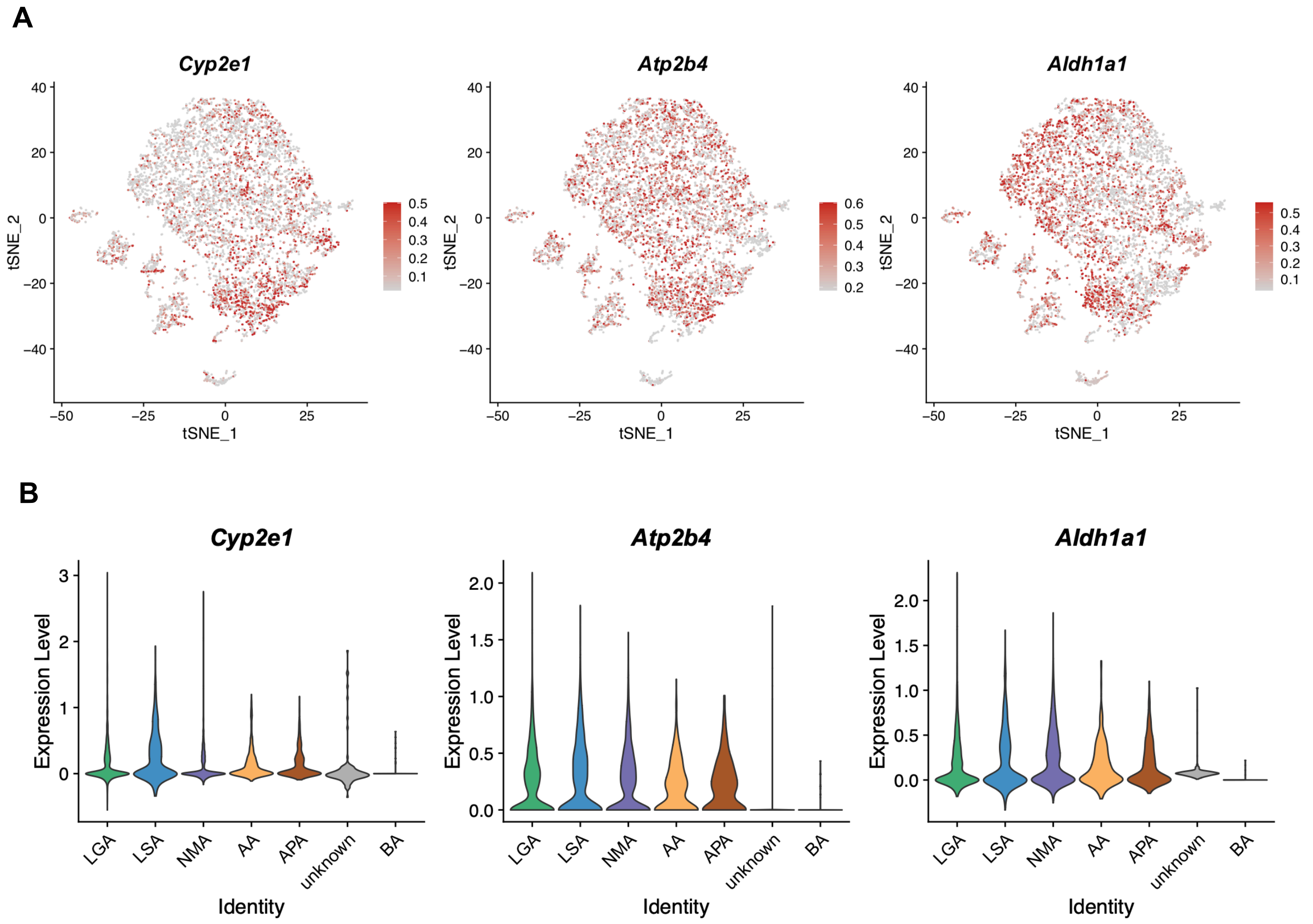

Figure S7. Marker genes for Cyp2e+ cells that negatively regulate brown adipocytes were excluded in beige adipocytes. (A) Feature plots of genes in adipocyte subclusters. (B) Violin plots of genes in adipocyte subclusters.

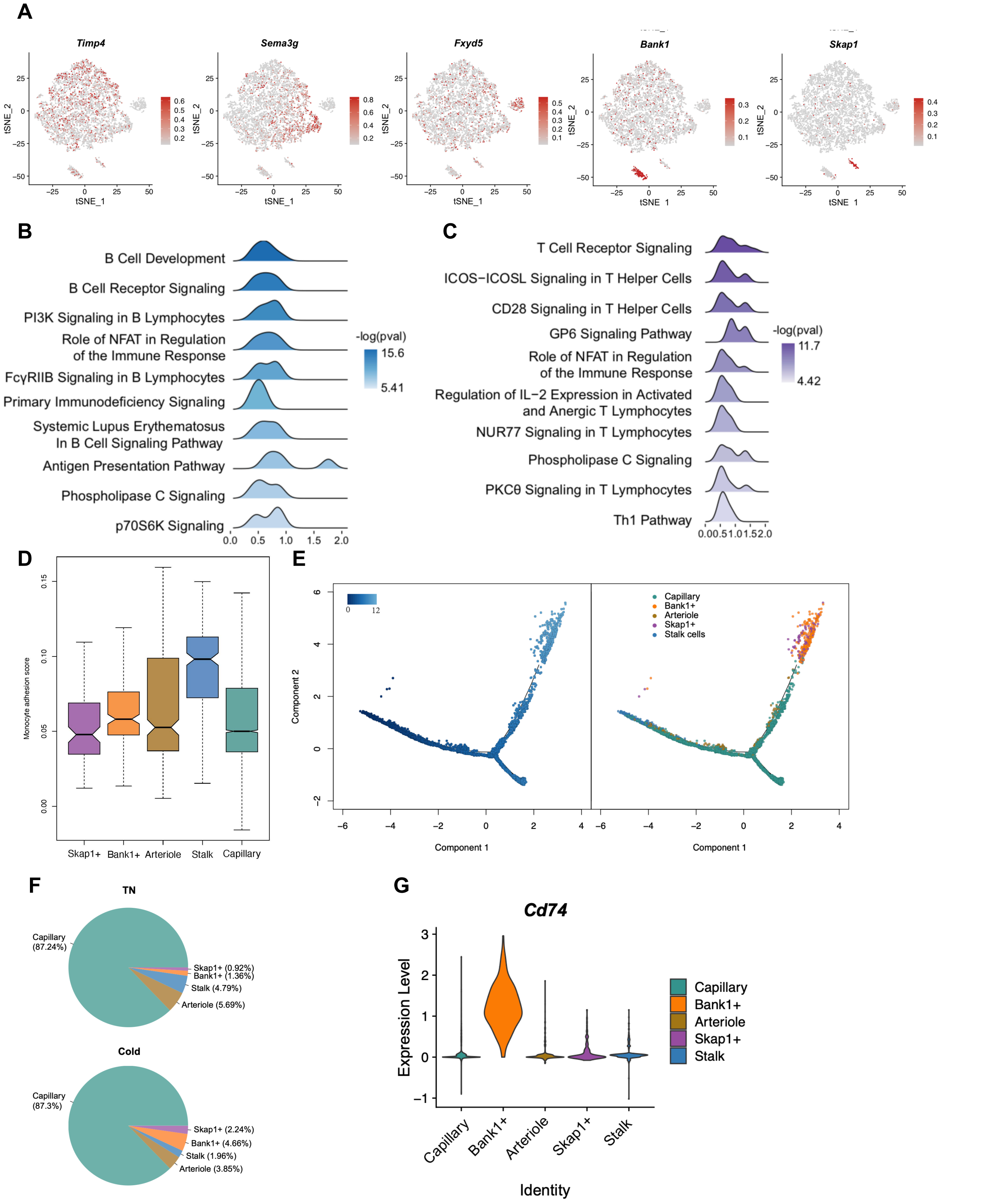

Figure S8. Characterization of endothelial cells in iWAT from TN and cold adapted mice. (A) Feature plots of marker genes in endothelial cell subclusters. (B)-(C) Top 10 most significantly enriched pathways of pseudo-time related genes in (B) Bank1+ and (C) Skap1+ endothelial cells. (D) Monocyte adhesion score. (E) The trajectory inference of all endothelial subclusters. (F) The fraction of each endothelial subpopulation. (G) VInplot of Cd74 in endothelial cell subclusters.

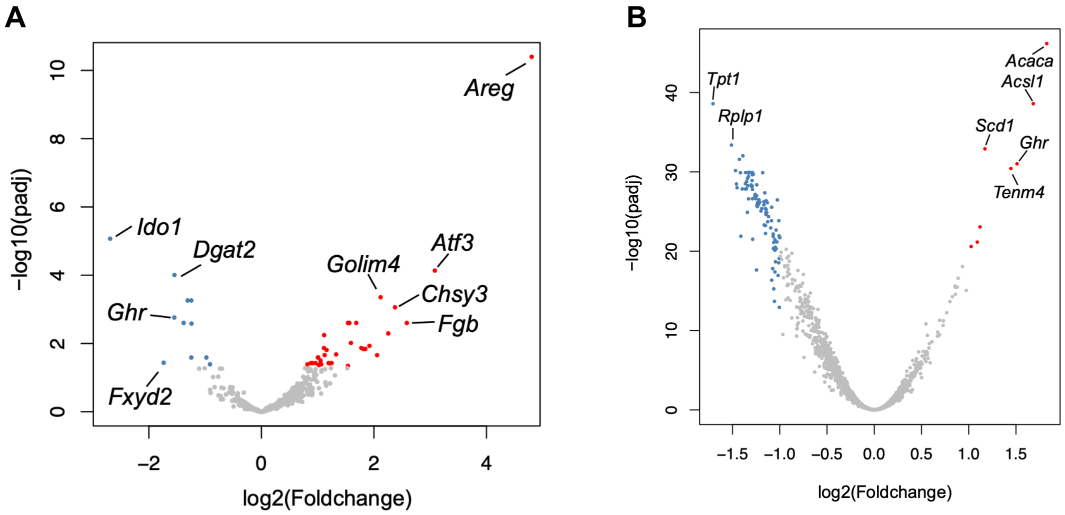

Figure S9. Gene expression was altered in iWAT schwann cells and SMCs by cold. (A) Volcano plot of gene expressions in schwann cells. (B) Volcano plot of gene expressions in SMCs.

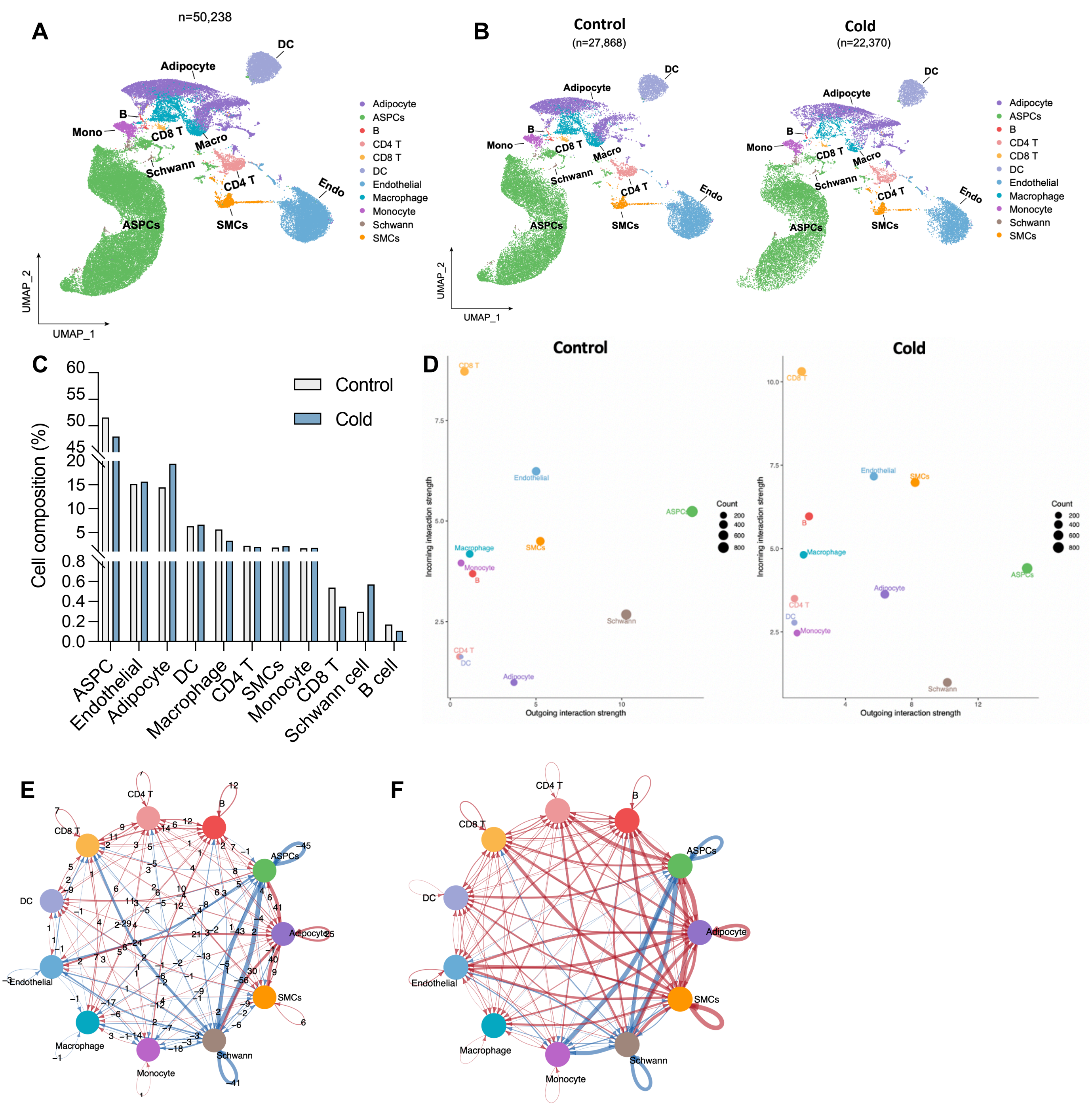

Figure S10. Integrative analysis of immune, non-immune cells and adipocytes in iWAT before and after cold exposure. The sc-seq dataset including the total stromal vascular cells in mouse iWAT with or without cold exposure was integrated with our data for further analysis. (A) UMAP of all the cell types. (B) UMAP of the cells grouped by cold and control conditions.(C) The percentage of the cell types under different conditions. (D)-(F) The cell-cell interactions within iWAT. (D) Overview of the number and the strength of the interactions between cell types. (E)-(F) The number (E) and the relative strength (F) of the unique intercellular interactions after cold exposure relative to the condition before cold exposure (Cold vs. Control). Red = increase, Blue = decrease.

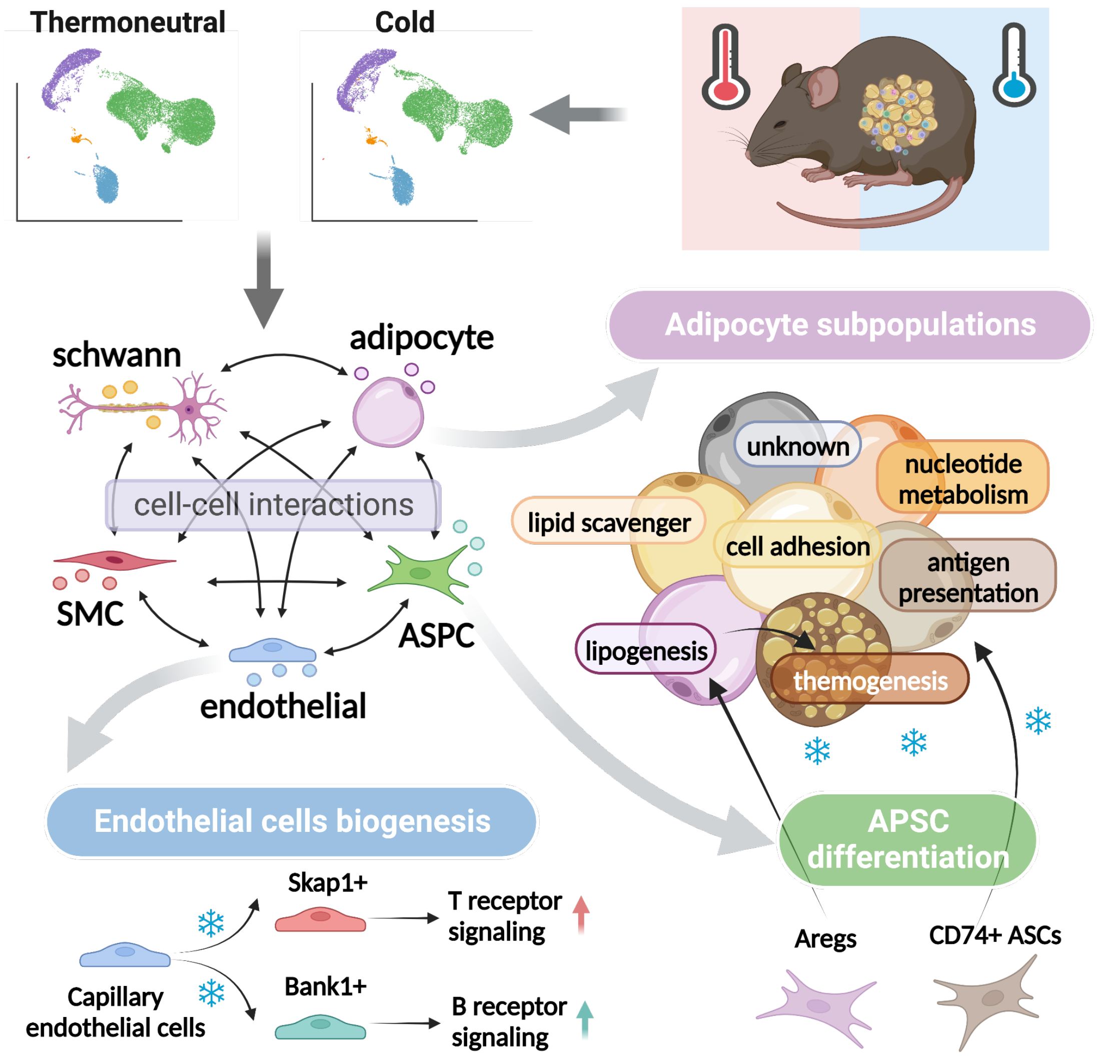

Figure S11. A single cell atlas consisting mature adipocytes and non-immune stromal vascular cells in mouse iWAT under thermoneutrality and after cold adaptation is depicted. Cell-cell communications, transcriptomic and secretomic profiles, are substantially remodeled in response to cold exposure. New subpopulations in adipocytes and endothelial cells, and the possible precursor cells are identified.
